# Repercussions of the COVID-19 pandemic on the HIV care continuum and related factors in economically disadvantaged nations: an integrated analysis using mixed-methods systematic review

**DOI:** 10.1186/s40001-024-01917-1

**Published:** 2024-06-26

**Authors:** Emmanuela Ojukwu, Ava Pashaei, Juliana Cunha Maia, Oserekpamen Favour Omobhude, Abdulaziz Tawfik, Yvonne Nguyen

**Affiliations:** 1 School of Nursing, University of British Columbia, Vancouver, BC Canada; 2 Federal University of Ceará, Fortaleza, Brazil; 3 New York Medical College, New York, USA

**Keywords:** HIV/AIDS, COVID-19, Continuity of patient care, Low-income countries, Systematic review, Barriers, Facilitators

## Abstract

**Background:**

The COVID-19 pandemic affected the self-management and care of people living with HIV, requiring adaptations in the way health services are provided. However, it is unclear how these changes impacted HIV care in low-income countries.

**Methods:**

A systematic review including the current evidence related to changes in HIV care continuum during COVID-19 was conducted through a systematic search in the online databases including CINAHL, OVID-Medline, CAB Direct, and OVID-Embase. A two-step screening process was carried out to include eligible papers and reports according to inclusion criteria.

**Results:**

From the searches we identified 21 total studies published between 2021 and 2024, the studies revealed mostly negative impacts on all stages of the HIV care continuum in low-income countries. There were impacts related to the blocking measures due to COVID-19, fear of contracting the disease, difficulties in providing resources such as income, food and transports, reductions in the provision of care from prevention to viral suppression.

**Conclusion:**

Overall, researchers identified several negative impacts of COVID-19 restrictions on HIV care continuum during pandemic; however, some observations indicated indirect positive impacts on some aspects of HIV care. Decline in HIV care practices during pandemic compared to before pandemic were observed including using preventative methods, counseling and testing, receiving HIV healthcare services, HIV medical appointments, antiretroviral adherence, engagement with treatment, and poor viral suppression. However, in some evidence improvement in ART adherence and PrEP use were observed.

**Supplementary Information:**

The online version contains supplementary material available at 10.1186/s40001-024-01917-1.

## Background

The outbreak of the Coronavirus Disease 2019 (COVID-19) pandemic directly caused high morbidity and mortality, as well as threatening general public health services and care delivery worldwide [1]. The severity of the COVID-19 pandemic has resulted in the health systems being overwhelmed and imposed lockdown measures to limit the spread of the virus in the community or region, leading to reduced access to health care [2, 3]. Evidence has shown that these measures have led to restrictions by healthcare facilities on the management of emergency medical conditions and chronic disease care and treatment services [4, 5].

The negative consequences of COVID-19 for some populations are more severe than others, mainly in populations of low-income countries (LIC), including job loss, food insecurity, an inability to manage existing conditions, and an inability to maintain preventive measures such as social distancing and use of personal protective equipment (PPE). Those living in poverty have less control over their living conditions and immediate environment, so the barriers they face in trying to protect themselves and their families are greater than those not living in poverty [6, 7]. Among the most underprivileged and disadvantaged in the era of pandemic are PLWH, people at risk of contracting HIV such as sex workers, people who inject drugs and men who have sex with men and people with other autoimmune diseases [8].

HIV prevention and care is critical to mitigating the public health threat of HIV and comprises HIV care continuum (HCC) measures, which consists of 5 steps, which are (i) diagnosis, (ii) linkage to care, (iii) retention in care, (iv) adherence to antiretroviral therapy, and (v) viral suppression [9–11].

The pandemic brought about significant changes in the provision of health services and increased fears about the increase in deaths and illnesses, health inequalities and the consequences of these changes among various subgroups of people living with HIV or at risk of contracting HIV. Containment measures, disruptions to supply chains and loss of income have the potential to exacerbate the impacts of the pandemic on HIV patients [12].

Many studies have reported a negative impact of the COVID-19 pandemic on HIV care access and delivery across different continents, highlighting the disruptions experienced primarily by PLWH [13–16]. However, similar studies exploring the impact of COVID-19 on HCC with a global focus, specifically on LIC are limited. It should be noted that a systematic review is an important resource for capturing quantitative and qualitative evidence, as it allows obtaining a holistic understanding of how HIV prevention and viral suppression were impacted by the COVID-19 pandemic, as well as the barriers and facilitators. This study aimed to understand the impact of the COVID-19 pandemic on HCC for people living with HIV or at risk of contracting HIV in low-income countries.

## Materials and methods

### Study design and setting

This research constitutes a systematic review conducted in accordance with the guidelines of the JBI mixed-methods systematic reviews (MMSR) methodology, initially undertaken in June 2022 and subsequently updated in January 2024. The systematic review was registered in PROSPERO (PROSPERO CRD42021285677); and a protocol which aided the study’s conduct was developed [17]. The authors tried to investigate the impacts of the COVID-19 pandemic on HCC in people living with HIV or at risk of contracting HIV in low-income countries. This study adhered to the Preferred Reporting Items for Systematic Reviews and Meta-Analyses (PRISMA) to ensure the reliability and validity of the study results.

This study is part of an extensive project examining the repercussions of the COVID-19 pandemic [17], including associated barriers and facilitators, across distinct country groups categorized by income levels—namely, high, middle, and low-income according to the World Bank Group classifications in the July 2022 [18]. Specifically, the current investigation hones in on low-income countries, as defined by the World Bank Group criteria for countries whose gross national income (GNI) per capita are less than $1085 USD. Comprehensive results for middle-income countries [19] high-income countries [20], along with comparisons across global income levels and multinational studies [21], have already been compiled and are readily accessible.

### Data sources

Our search strategy was developed in consultation with a subject expert librarian at the University of British Columbia to locate both published and unpublished studies. Multiple variations and synonyms of the index terms “HIV” and “COVID-19” were searched in CINAHL (EBSCOhost), MEDLINE (Ovid), CAB Direct, and Embase (OVID). Using the COVID-19 special filter in OVID-Embase, a refined search approach was used to comb through databases including CINAHL, OVID-Medline, CAB Direct, and OVID-Embase. The COVID-19 special filter was used in Embase (Ovid), and a sample search strategy is presented in **Appendix I**. In addition, references in articles selected for full-text review were manually reviewed to identify further citations that match the criteria of this review.

### Study selection

The first search was done in CINAHL using the keywords "HIV" and "COVID-19", with relevant terms chosen from the titles and abstracts of pertinent studies. The reference lists of the chosen papers were also personally reviewed. The search encompassed papers published between March 2020 and January 2024. Moreover, the studies included were primarily in English; studies in a foreign language were translated by the reviewer and either included or excluded depending on suitability.

Duplicate records were automatically removed from all uploaded retrieved citations in Covidence. The titles and abstracts were evaluated by two separate reviewers, who removed those that didn't fit the requirements for inclusion. Two reviewers read selected citations in full, with the grounds for exclusion being recorded in Covidence. Discussion or the participation of a third reviewer was used to settle disagreements among reviewers. A flowchart detailing the screening procedure was created in accordance with PRISMA-ScR recommendations for openness and reproducibility.

The exclusion criteria: duplicate studies, conference articles, articles with unavailable full texts or gray literature, articles not related to HCC, intervention studies or studies that did not have a design suitable for the objectives of this review.

### Data extraction

Both quantitative and qualitative data were extracted from studies included in the review by 2 independent reviewers using a self-developed extraction tool. When necessary, the data extraction tools were modified to accommodate the differences of each included study, and modifications were detailed in the systematic review. The data extracted included specific details about the populations; study methods; theoretical framework, where applicable, phase of HCC; context; and outcomes of relevance to the review question, including the implications for clinical practice. Specifically, quantitative data were composed of outcomes of descriptive and inferential statistical tests. In addition, qualitative data were composed of verbatim themes or subthemes with corresponding illustrations and will be assigned a level of credibility. Any disagreements that arose between the reviewers were resolved through discussion or with an additional third reviewer. Where necessary, authors of papers were contacted to request missing or additional data.

### Quality assessment

Multiple critical appraisal tools were used because a variety of study designs were included. While qualitative studies were evaluated using the JBI critical appraisal tool for qualitative research, quantitative studies were evaluated using JBI critical appraisal tools for different study types. The methodological quality of research reports and pre-print publications were evaluated. The appraisal was conducted independently by two reviewers, with any disputes being settled by discussion or the participation of a third reviewer. Studies that didn't fulfill the minimum standards of quality were not considered. The results of Risk of Bias/Quality Assessment were placed into categories based on the sum of points given by each reviewer (Low Risk of Bias [LRB], Medium Risk of Bias [MRB], High Risk of Bias [HRB]).

The review used Covidence and a convergent integrated approach in accordance with the JBI methodology for MMSR. While qualitative data were combined with the quantitative data, qualitative data from quantitative studies were turned into textual descriptions or narrative interpretations. The integrated findings were presented as line of action statements, and similarity in meaning were noted.

## Results

**Fig. 1:**
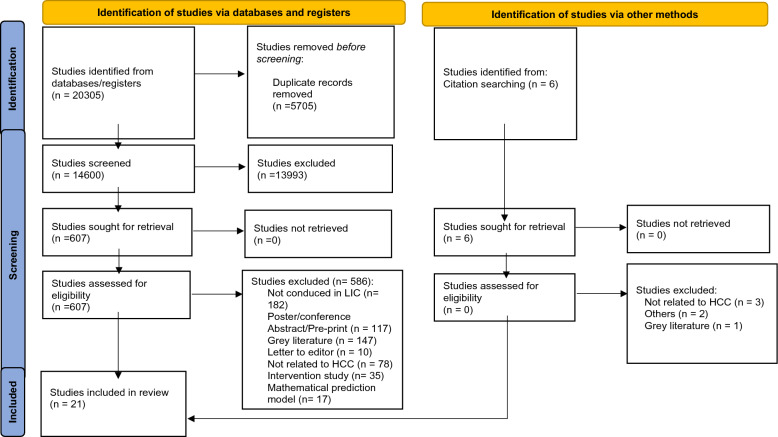
PRISMA 2020 flow diagram

After conducting an initial search of online databases according to search strategy, a total of 20,305 records were obtained; after removing 5705 duplicates, further analysis was done with 14,600 unique records. The initial screening of title and abstract was done by two independent researchers. In the case of any disagreement, the third researcher was consulted to make the final decision on whether to include or exclude the articles. After exclusion of 13,993 articles, full text of 607 articles were reviewed. Finally, we identified 21 eligible articles for data extraction. The selection process of articles were shown in PRISMA diagram (Fig. 1). The quality of the included articles was assessed by two independent researchers. All assessed articles had low risk of bias by attaining 65% or higher of quality assessment (QA) score.

**Fig. 2:**
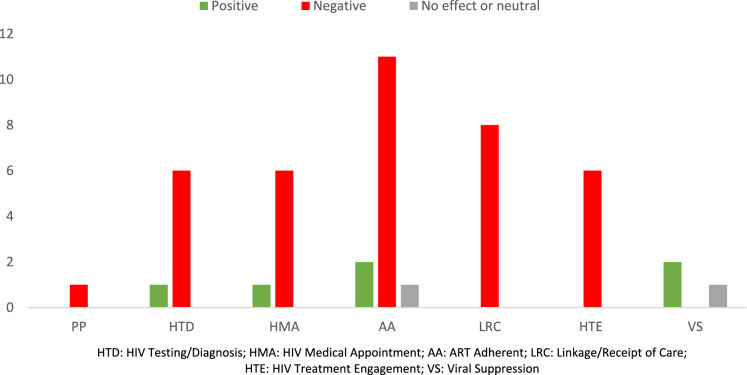
Distribution of papers by the stage(s) of the HIV care continuum in low-income countries

The study population in all included studies was around 696,746. Gender distribution was not reported in all studies. Regarding the year of publication, 8 studies have been published in 2021, 7 studies in 2022, 4 studies in 2023, 1 study in 2024, and 1 study in 2020 (Table 1 and Fig. 2 illustrate a summary of the findings).

**Table 1:** Comprehensive details of the included articles

	Author	Country and year	Setting	Sample size	Results		Barriers	Facilitators
					Stage of care continuum	COVID impact on HCC stage		
1	Adugna [23]	Ethiopia 2021	HIV care facilities, health centers, hospital	51,990	HTD, AA	**HTD:** 37.9% decrease from pre-COVID to lockdown and a 25.4% decrease from the lockdown period to post-lockdown in VCT attendants; 32% decrease from pre-COVID to lockdown and a 46% increase from the lockdown period to post-lockdown in Provider-initiated counselling and testing attendants **AA:** 20.2% decrease from pre-COVID to lockdown and a 22.5% increase from the lockdown period to post-lockdown in mean ART attendants. Before COVID-19, the number of newly started ART in May 2019 was 49 but during lockdown, it was reduced to 15	Fear of contracting COVID, COVID infections, lockdown and quarantine measures	NA
2	Tolossa [38]	Ethiopia 2021	Public health facilities	361	AA	**AA:** 78.7% had good adherence, while 21.3% had poor adherence	Living in a rural region, being young, using drugs, COVID-19 era's lack of infrastructure, staff shortages, inability to refill ART due to restrictions	NA
3	Wagner [39]	Uganda 2021	HIV clinic	14,632	AA	**AA:** the percentage of days without a personal ART supply after lockdown remained high, standing at 14% of days. It was 80% before lockdown	Quarantining and social segregation, travel restrictions, food insecurity	N/A
4	West [25]	Uganda 2022	Health sciences program clinics	16 PLHIV, 10 Health workers	AA	**AA:** early on in pandemic, access to clinics was hampered by transportation limitations, which raised concerns about ART shortages and increased anxiety	Fear of judgment over HIV status, transportation restrictions due to COVID, inflated transportation costs	NA
5	Linnemayr [14]	Uganda 2021	Clinic	100	AA, LRC, HTE	**LRC, HTE:** 76% COVID-19 negatively impacted travel to HIV clinics; 54% perceived that coming to the clinic increased their risk of acquiring COVID-19; **AA:** 14% COVID-19 had negatively impacted their ART adherence	Limited transportation, fear of COVID infection	NA
6	Muwanguzi [30]	Uganda 2021	Private security companies	44	LRC, HTE	**LRC:** several participants who had recently started on ART were unable to continue treatment HTE: a few participants experienced interruptions in treatment schedule	Limited transportation, COVID-19 measures (stay-at-home directives), stigma/fear of partner	NA
7	Thekkur [24]	Malawi 2021	Referral hospital, secondary referral hospital-specific for HIV/AIDS and TB, community hospital, health centers	NA	HTD	**HTD:** 39% decrease in the number of persons who underwent HIV testing from pre-COVID to during COVID-19	Transportation difficulties, as well as community fear of health facilities, were thought to hinder health facility access	NA
8	Palattiyil [34]	Uganda 2022	Health centers, NGOs: AIDS Information Centre and Africa Humanitarian Action	229	HMA, AA	**HMA:** 52% without access to community outreach had challenged to receive TB or HIV/AIDS services; **AA:** difficulty in getting medications	Public transportation restrictions, expensive transport expenses	Reduction in long lines and hours of waiting, administration of medications via VHTs, prolonged dosing regimens
9	Nalubega [29]	Uganda 2021	Peri-Rural and rural healthcare facilitates	17	LRC	**LRC:** reengagement and retention in care negatively impacted	Structural (stigma due to home delivery of ART), clinical (fear of getting COVID-19, food insecurity due to pandemic), psychological (fear of HIV stigma, ART interruption to keep HIV status as a secret from their spouses during the lockdown at home)	Home delivery of HIV medicine
10	Wagner [37]	Uganda 2020	Clinic	280	AA	**AA:** 8.9% significant decrease in ART adherent	Restricted public transportation, elevated depressive symptoms, food insecurity	Improving mental health, depression treatment
11	Chilot [28]	Ethiopia 2021	Healthcare facilities	212	HMA, HTE, VS	**HTE:** 58 (27.4%) missed refill visits, **VS**: 56 (26.4%) missed follow-up diagnostic test, **HMA:** 55 (26%) missed counseling services	Age ≥ 55, fear of COVID-19, age ≥ 55, transport disruption, high cost of traveling to Healthcare facilities, limited access to mask and sanitizer, partial lockdown	NA
12	Parmley [22]	Zambia 2023	Key population civil society organizations	60	PP, HTD, AA, HTE	**PP**: all participants reported reduced supply of condoms; **HTD:** all participants reported HIV testing barriers, also benefit from self-testing was positive impact; **HTE:** evasion of seeking care for HIV, also benefit from mobile clinics; **AA:** depletion of ART inventory	Fear of contracting COVID-19, lockdowns	Self-testing, mobile clinic, appointment reminders
13	Kabami [31]	Uganda 2023	Electronic medical records	7071	LRC, HMA, VS	**LRC:** decline in ART initiation; **HMA:** less than 10% missed visits; **VS:** no significant difference	Lockdown	NA
14	Mupambireyi [40]	Zimbabwe 2024	Clinic	20	HTE	**HTE:** limited utilization of HIV care and prevention programs	Fear of contracting infection or putting families at risk. fear of COVID-19 and unintentional disclosure of HIV status, financial concerns, clinic closures, staff shortages, reduced operating times, and limitations on the number of patients seen per day	NA
15	Kalua [41]	Malawi 2022	Dataset of HIV clinics	556,281	HTD	**HTD:** 40% decline in VL testing	COVID-19 restrictions	NA
16	Shimels [32]	Ethiopia 2023	Public health facilities	371	LRC, AA	**LRC:** 19% challenges in follow-ups; **AA:** 13.5% availability of medications, 54% perfect ART adherence	COVID-19 restrictions	Having basic education, being married, attending to a health center, and having sleep disturbance
17	Emmanuel [51]	Uganda 2022	Dataset of HIV clinics	9952	AA	**AA:** 14.7% had inadequate ART utilization	Obesity	Community-focused approaches, ensuring Continuity of HIV Clinic Services Amidst the Pandemic, phone follow up of those missed their appointment
18	Lakoh [26]	Sierra Leone 2023	Public health facilities	8538	LRC, HTD	**HTD:** 41.2% and 35.7% decline in HIV testing services; **LRC:** linkage to care was higher than before pandemic	Fear of COVID-19 exposure, restricted access to services, and disruptions to social life and livelihoods	NA
19	Shah [33]	Democratic Republic of the Congo 2022	HIV clinics	36,585	LRC	**LRC:** 5% reduction in initiation	NA	Lockdowns possibly kept patients closer to HIV clinics, altered ART care models enabled wider access to multi-month dispensing, benefitting various age groups
20	Izudi [27]	Uganda 2022	Electronic medical records	9952	HTD, VS, LRC, HTE	**HTD:** 7% decrease in viral load testing coverage; **VS:** 1% improvement in viral load suppression; **LRC:** in the comparison group, significantly lower percentage (33.1%) underwent viral load testing compared to the exposed group (44.2%); **HTE:** only 19.1% of participants retained in care	COVID-19 lockdown	Nationwide guidelines for sustaining HIV services during restrictions; the Presidential Emergency Plan for AIDS Relief; multiple service delivery models for distributing HIV medications
21	Mukamba [36]	Zambia 2022	Healthcare facilities	25	AA, HMA, HTE	**AA:** reduction in ART adherence, also consistency in taking ART; **HTE:** decrease in seeking HIV care; **HMA:** decrease in hospital visits for HIV-related care	COVID-19 restrictions, bad attitude of healthcare workers, stigma, drug side effects, food insecurity	Long ART delivery service

### Prevention/PrEP use (PP)

Parmley et al.'s study was the sole investigation that examined the impact of the COVID-19 pandemic on the PP stage. In their study, all participants reported experiencing a reduced supply of condoms due to the pandemic [22].

### HIV testing/diagnoses (HTD)

Six studies have highlighted the adverse impact of the COVID-19 pandemic on HTD [23–28], while one study also noted some positive effects [25]. A study from Ethiopia came from an institution-based, repeated cross-sectional study with a sample of 51,990 reported a decline in voluntary and provider-initiated counseling and testing, between the pre-COVID and lockdown period. While voluntary counseling and testing continued to decline from the lockdown to post-lockdown period, there was a large increase in provider-initiated counseling and testing within the same period [23]. In a study conducted in Malawi HIV program services were monitored in eight health facilities. The authors reported a substantial decrease in the number of people who underwent HIV testing, from the pre-epidemic to pandemic time periods [24]. A study conducted in Sierra Leone among 8538 PLHIV revealed a 41.2% decline in HIV testing services during the early phase of the COVID-19 pandemic compared to pre-pandemic levels. Additionally, there was a 35.7% decline observed between 2019 and 2021 (late intra-COVID-19) [26]. Two studies conducted in Uganda reported a decrease in HIV testing rates [25, 27]. However, one of these studies highlighted the positive impact of self-testing, demonstrating its benefits [25]. Some possible reasons for the decline in HIV testing may be due to the fear of contracting COVID-19 and the resultant lockdown and quarantine measures, such as curfews and travel restrictions. Regarding facilitator factors, studies have highlighted the provision of self-testing and mobile clinics.

### Linkage/receipt to care (LRC)

Eight studies documented negative impact of the pandemic on LRC [14, 26, 27, 29–33]. Research conducted in low-income countries has provided evidence indicating the existence of various impediments to accessing and receiving necessary healthcare services, referred to as LRC, during the COVID-19 pandemic [29]. These obstacles can be categorized as structural, clinical, and psychological. Structural barriers encompass factors such as long distances between individuals and healthcare facilities, reduced income resulting from unemployment, and challenges related to taking medications without consuming food. These structural barriers have hindered the ability of PLWH to engage in LRC. Similar findings were reported in another study, which revealed that 76% of PLWH experienced difficulties in reaching healthcare clinics due to the COVID-19 pandemic [14].

On the clinical front, PLHW exhibited concerns about the risk of contracting COVID-19, leading to their reluctance in attending clinical appointments. Correspondingly, another study found that 54% of PLWH perceived an increased risk of COVID-19 infection when attending appointments at HIV clinics. Finally, psychological barriers emerged, primarily related to the intensified stigma surrounding HIV during the COVID-19 pandemic [29]. The limited resources and staffing shortages resulting from financial constraints have contributed to decreased appointment availability, longer wait times, and reduced quality of care in healthcare settings. These challenges have further impeded the ability of PLWH to access the necessary services and support for managing their condition effectively.

### HIV medical appointments (HMA)

There were five studies from low-income countries which looked at the impact of COVID-19 on HIV medical appointments and all of them reported negative impact [28, 31, 34–36]. A cross-sectional study from Ethiopia which surveyed 212 adults, including 63% female, reported that about a quarter of participants had missed counselling services due to the pandemic [28]. A mixed-methods study from Uganda utilized a survey and in-depth interviews to analyze HIV care across four health centers. The authors noted that over half of participants were unable to access community outreach and subsequently challenged to receive HIV/AIDS services [34]. Some barriers to HIV medical appointments were the fear of contracting COVID-19 and pandemic measures, such as travel restrictions and lockdowns. The high cost of traveling to health facilities and limited access to PPE could have also resulted in poor HMA attendance.

### ART adherence (AA)

The majority of studies from low-income countries focused on changes in ART adherence due to the COVID-19 pandemic. Among them, 12 studies reported negative effects, one of which also noted a positive impact, while another reported no changes. Adugna et al. in a cross-sectional study identified a 20.2% reduction in patients on ART when comparing the pre-pandemic periods and during the pandemic, there was also a reduction in the number of new patients who started ART. The fear of contracting COVID-19, lockdown and quarantine measures were cited as barriers [23]. Palattiyil et al. found negative effects for adherence to antiretroviral therapy were related to the difficulty of obtaining medications, barriers related to public transport restrictions and high transport costs, which affected the lack of consultations and patient follow-up. The lack of basic provisions, such as food, as well as the lack of social support and stigma, were factors that made it difficult to take ART. As facilitators, the reduction of queues for care, the delivery of medicines by health agents and the increase in dosage regimens, which were adjusted to reduce the number of trips to obtain the medication, were listed [34]. Adverse effect on AA from lack of financial resources, lack of food and stigma was also reported, with 14% of respondents reporting that COVID-19 had negatively impacted AA. The fear of becoming infected with COVID-19 discouraged attendance at health facilities, as well as a lack of medication and uncertainty as to which HIV clinic would be available to provide care [14]. Wagner et al. identified that elevated symptoms of depression were also associated with lower adherence to antiretroviral therapy during the lockdown period. Participants indicated that the pandemic contributed to their heightened symptoms of depression through various economic-related stressors, including lack of food, work, and money [37]. Another cross-sectional study found that 21% reported missing picking up HIV medicine, 12% missing taking a dose of HIV medicine, and 11% stopping taking HIV medicine. In multivariate analysis, higher COVID-19 related mental health challenges and COVID-19 related financial concerns were related to greater likelihood of reduction in HIV care [35]. Tolossa et al. found that 21.3% of participants had poor adherence to ART, with 3.37 times greater odds of poor adherence among people residing in rural areas and 3.41 times greater in patients aged less than 35 years. Substance use was strongly associated with poor AA, with 5.42 times greater odds of poor adherence among users [38]. The cohort of Wagner et al. showed an increased risk of running out of personal ART supply, with 11.2% reporting that the pandemic reduced their ability to adhere to medication and 8.3% with worsening adherence during the lockdown. It is noteworthy that there was an increase in the percentage, which corresponded to 12.1% of people who stated that they missed a dose of ART due to lack of food. The authors believe that a facilitator for maintaining treatment was the existence of personal stocks of previous prescriptions, which ensured a sufficient supply of antiretroviral drugs [39]. West et al. found that at the beginning of the pandemic, access to clinics was hampered due to transport limitations, which raised concerns about shortages of ART and increased anxiety [25]. Two studies published in 2023 highlighted interruptions in ART adherence attributed to the depletion of ART inventory [22] and unavailability of medications [32].

The main barriers cited as obstacles included the fear of contracting COVID-19, lockdown and quarantine measures, restrictions on public transportation, and high transportation costs. Additional barriers identified included increased mental health challenges, such as symptoms of depression, and the fear of being judged based on HIV status. Financial instability, food insecurity, and the negative impact on pill-taking practices also posed challenges, as they disrupted the structure and daily routines necessary for consistent adherence. Regarding health services, one study specifically highlighted that inadequate infrastructure during the pandemic, staff shortages, and limitations on home visits further contributed to low ART adherence. On the other hand, certain factors have been identified as facilitators for ART during the pandemic, including modifications to the ART care model, home delivery of ART, multi-month dispensation of ART. Also, mental health support interventions aimed at improving mental well-being and addressing depressive symptoms.

### HIV treatment engagement (HTE)

Findings from eight researches showed that both quantitative and qualitative data collectively emphasize the adverse effects of COVID-19 on treatment engagement within the HIV care continuum in low-income countries, including missed refill visits, perceived impact on care and treatment, interruptions in treatment schedules, and compromised access to specialized services. [14, 25, 27, 28, 30, 35, 36, 40]. Only one study among them also reported a positive effect [25].

Quantitative data from a cross-sectional study at Ethiopia, by Chilot et al., with 212 participants revealed that 27.4% of individuals missed their refill visits, indicating disruptions in accessing necessary medications [28]. Additionally, Wang et al., in a cross-sectional study in the Dominican Republic, 187 female sex workers, reported that 34% of individuals stated that COVID-19 had an impact on their HIV care and treatment [35]. Qualitative findings by Muwanguzi et al., in prospective interviews with 44 men, at Uganda, highlighted few participants who experienced interruptions in their treatment schedules [30].

A mixed-methods study by Linnemayr et al. conducted in Uganda, with 100 participants, had quantitative findings from descriptive statistics, which demonstrated that 76% of participants reported negative effects on their ability to travel to HIV clinics, while 54% perceived an increased risk of acquiring COVID-19 by attending the clinics [14]. Qualitative results were obtained through transcription and analysis of interviews with participants. This portion of this study identified themes including the impact of COVID-19 lockdowns on clinic attendance, concerns about exposure at clinics, effects on antiretroviral therapy adherence, perceptions of susceptibility to COVID-19, and strategies to reduce risk and increase resilience. These insights emphasize the challenges faced in accessing and engaging in HIV care during the pandemic, highlighting the need for targeted interventions to maintain continuity of care in low-income settings.

As for the barriers mentioned in studies that evaluated the impact of the COVID-19 pandemic on HTE, barriers commonly mentioned included individuals being over 55 years old, fear of contracting COVID-19, limited access to personal protective equipment like masks and sanitizers, difficulty in accessing healthcare services, and insufficient funding for services tailored to adolescents. Other significant obstacles involved transportation disruptions, high costs associated with traveling to healthcare facilities, lockdown measures, and stay-at-home directives. Additionally, financial concerns stemming from COVID-19, mental health challenges, emotional abuse from partners, partner stigma or fear, and challenges related to family planning were also reported as barriers. It is worth noting that no facilitators were identified in the studies addressing this phase of the HCC.

### Viral suppression (VS)

The COVID-19 pandemic positively affected viral suppression along the HIV care continuum in a 2022 study by Kalua et al. from Malawi. The cohort study conducted among 556,281 people found that viral suppression rate increased slightly during COVID-19 pandemic, rising from 93% pre-COVID-19 to 94% during COVID-19 [41]. Similar finding of 1% improvement in VS was reported in qualitative study conducted among 9952 PLHIV in Uganda [27]. A study reported no significant changes in VS during pandemic [31].

Facilitators for sustaining VS during restrictions include the implementation of nationwide guidelines, support from initiatives like the Presidential Emergency Plan for AIDS Relief (PEPFAR), utilization of multiple service delivery models for medication distribution, as well as factors such as older age, longer time on ART, being women.

## Discussion

Understanding HIV interaction as a life-long lasting infectious disease with other infectious diseases, especially those causing pandemics such as COVID-19, can provide a roadmap for care planning and management during the next pandemics. This study constituted a comprehensive examination of the interaction of HIV and COVID-19 pandemic in low-income countries in a period of approximately four years. Research findings have elucidated that although the pandemic had a discernible impact on healthcare services across a spectrum of diseases, individuals with chronic conditions, notably those who are HIV-positive, exhibited a heightened vulnerability [42–44].

We identified negative impact of COVID-19 on preventive methods. Limited access to condom due to concentration of HIV services on COVID-19 led to increased engagement in condom less sex in MSM/TGW. This finding indicates the importance of availability of HIV-related healthcare services during pandemics to access preventing methods in case of high-risk sexual behaviors. Efforts to provide condoms and prevention methods should prioritize diverse distribution channels, alongside integrating condom provision into COVID-19 service delivery models.

The diverse impacts of the COVID-19 pandemic on HIV testing services were observed. While some observations noted a decrease in testing rates, others highlighted nuanced shifts in testing practices. Factors such as fear of COVID-19 contraction and associated lockdown measures, including curfews and travel restrictions, likely contributed to the decline in testing. However, the emergence of self-testing methods [22] presented a potential avenue for maintaining testing accessibility amidst pandemic-related disruptions to traditional services. These findings underscore the need for tailored interventions to address barriers to HIV testing and ensure continuity of care within the context of ongoing public health crises. This finding aligns with a study conducted in a high-income country, indicating that the impacts of COVID-19 extend beyond economic status [45].

The pandemic period had deleterious repercussions on ART adherence and the commencement of pharmaceutical treatment in low-income countries. Potential impediments to adherence included impediments such as blockades and lockdown measures enforced due to quarantine restrictions and heightened apprehensions regarding COVID-19 transmission. Moreover, contributory factors encompassed a paucity of social support, the pervasive stigmatization of HIV, depressive states, substance misuse, obstacles in procuring medication, transportation-related challenges, and the exorbitant costs associated with public transportation. Additionally, concerns arose pertaining to fundamental resources such as sustenance, financial means, and employment. Several studies reported compromised functionality of HIV care facilities and anxieties regarding the availability of pharmaceutical drugs. In the context of resources aimed at enhancing antiretroviral adherence, the following strategies were identified: the provision of extended medication supplies [36], the home delivery of antiretroviral drugs [29], the establishment of individual antiretroviral stockpiles [33], and the option to adhere to medication regimens while remaining at home. These measures facilitated the establishment of a structured routine for adherence to medication protocols.

Regarding engagement with treatment, there were evident negative repercussions observed in the form of missed follow-up appointments and medication refill replacements, which had a detrimental impact on HIV care overall. Concurrently, there were shortcomings in the care provision by healthcare teams in HIV care services. The pandemic also hampered various sectors that, in turn, hindered the engagement of PLWH in their care. These challenges encompassed difficulties related to travel to healthcare clinics and the impracticability of available transportation means. Barriers to clinical care included concerns about attending face-to-face appointments and the heightened risk of COVID-19 exposure. Disruptions in transportation, partial lockdowns impinging on mobility, and income reductions all played pivotal roles in individuals' inability to access healthcare appointments, corroborating the findings of earlier research [46, 47] and aligning with analogous circumstances observed globally [30, 48–50]. These studies affirm that COVID-19 containment measures significantly curtailed patients' capacity to avail themselves of healthcare facilities. The findings pertaining to healthcare engagement reveal adverse effects on healthcare continuum compliance and underscore inadequacies in the methods employed for healthcare delivery and follow-up. These shortcomings are imperative to address in order to surmount the identified barriers experienced during the pandemic period in low-income countries.

Viral suppression was poorly reported as a phase of HCC impacted during the pandemic. There may have been a beneficial impact on virus suppression rates during the epidemic, according to reports. Despite the challenges posed by the pandemic, a combination of factors including the implementation of national guidelines, support from international programs like PEPFAR, and the utilization of diverse service delivery methods for medication distribution have likely contributed to the maintenance of viral suppression. This study highlights the significant positive impact of models such as PEPFAR on achieving viral suppression. PEPFAR's holistic approach, which encompasses ensuring access to antiretroviral therapy, adapting service delivery to current needs, strengthening health systems, fostering community engagement, and addressing health disparities, has played a pivotal role in advancing viral suppression efforts. By providing a detailed account of PEPFAR's contributions to promoting viral suppression, valuable insights can be gleaned for replication in various settings, thereby optimizing health services and improving outcomes for individuals living with HIV/AIDS. It's essential to acknowledge that the pandemic has affected different areas and populations unevenly, and variations in healthcare resources and access may have influenced outcomes. To fully grasp the dynamics of viral suppression throughout the pandemic and to identify effective strategies for enhancing the provision of HIV care during emergencies, additional research is necessary.

## Conclusion

Overall, researchers have discerned numerous adverse effects of COVID-19 restrictions on the HIV care continuum during the pandemic. In comparison to the period before the pandemic, a decline in various aspects of HIV care practices has been noted, encompassing the use of preventive measures, counseling and testing, the receipt of HIV healthcare services, attendance at HIV medical appointments, antiretroviral adherence, engagement with treatment, and suboptimal viral suppression. Nevertheless, certain observations have pointed to indirect positive impacts on specific facets of HIV care as a result of the implementation of condom distribution, self-testing services, extended medication supplies, and the home delivery of ART medications during the pandemic. Further research is needed to understand and address the disparities in healthcare access during emergencies.

## Limitations

The limitations that may compose the review are due to the small number of articles identified, despite having started from a broad search in various databases, the limited number reflects the impact on low-income countries, but as only a limited cut in the studies identified. If there were other studies in more low-income countries it would be beneficial to implement the big picture.

### Supplementary Information

Supplementary Material 1.

## Data Availability

The study has no additional data or materials to share.

## Abbreviations

AA: ART adherence
ART: Antiretroviral treatments
COVID-19: Coronavirus disease 2019
GNI: Gross national income
HCC: HIV care continuum
HMA: HIV medical appointments
HTD: HIV testing/diagnoses
HTE: HIV treatment engagement
LIC: Low-income countries
LRC: Linkage/receipt to care
MMSR: Mixed-methods systematic reviews
PLWH: People living with HIV
PPE: Personal protective equipment
PRISMA: Preferred Reporting Items for Systematic Reviews and Meta-Analyses
QA: Quality assessment
